# Supplementary material for: Recovering High‐Quality Host Genomes from Gut Metagenomic Data through Genotype Imputation
Source: Adv Genet (Hoboken). 2022 May 6;3(3):2100065. doi: 10.1002/ggn2.202100065 (PMC9744478; doi:10.1002/ggn2.202100065)
Supplement: Supplementary file 1 — Supporting Information [file GGN2-3-2100065-s006.pdf]

ADVANCED GENETICS
Open Access

Supporting Information

for *Advanced Genetics*, DOI 10.1002/ggn2.202100065

Recovering High-Quality Host Genomes from Gut Metagenomic Data through Genotype Imputation

Sofia Marcos, Melanie Parejo, Andone Estonba and Antton Alberdi**

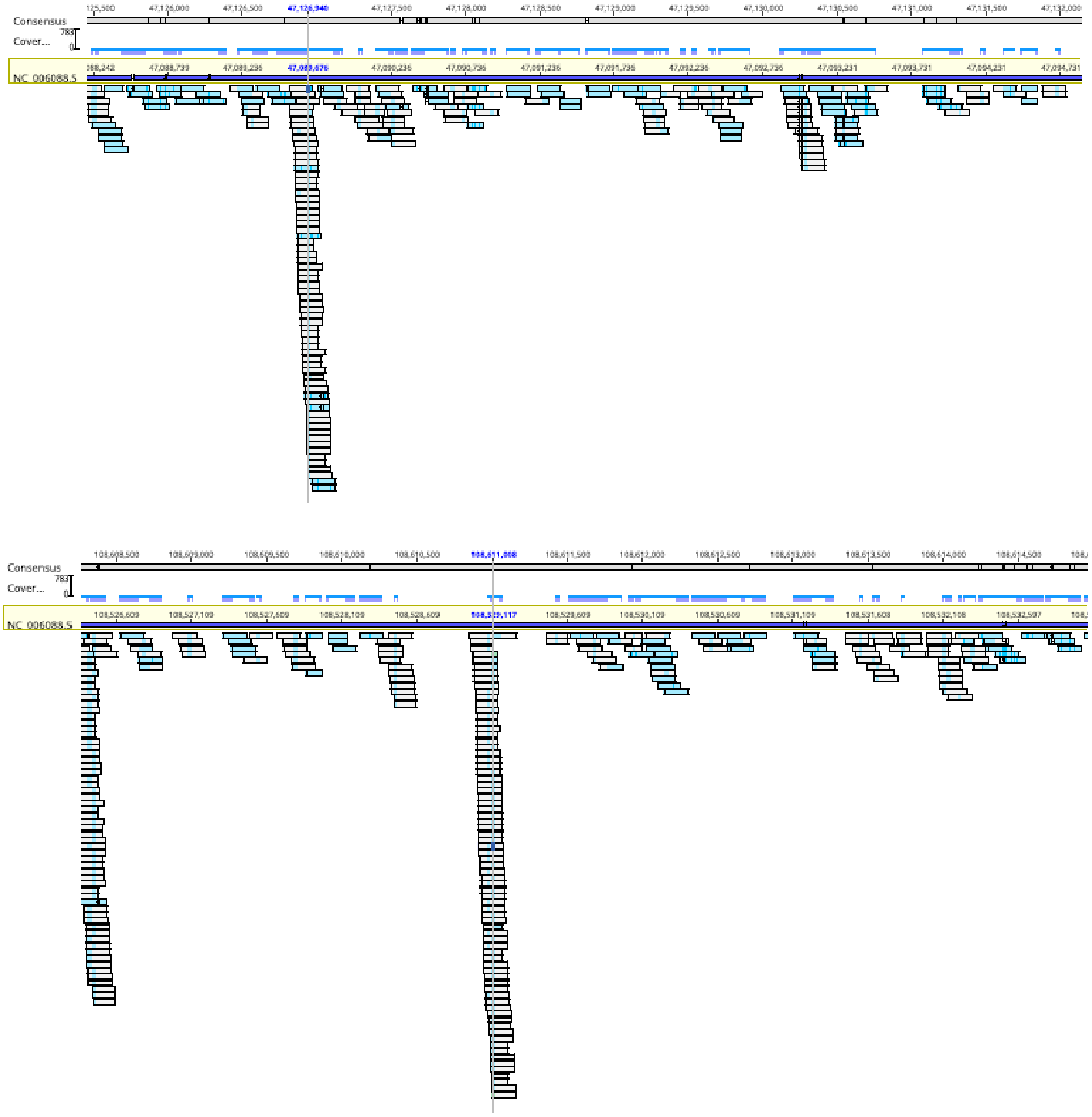

Figure S1. Mapped reads distribution after performing alignment with standard parameters (seed length 19). Captures from 47 and 180 Mbp regions from chromosome 1 with Geneious.

Table S1. Mapping depth and breadth results before and after changing seed length from 19 to 25.

Parameters	K19	K25
Depth	2.78	1.73
Std. depth	202.79	3.66
Breadth	56.74	51.60
Insert size	20	179

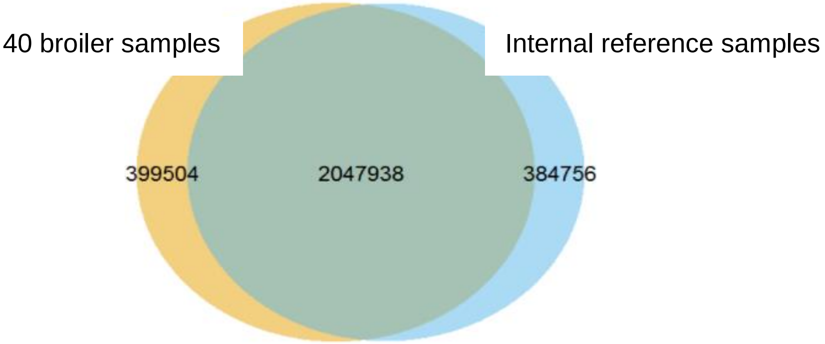

Figure S2. Venn diagram of shared variants between the internal reference samples and the variant called 40 broilers of the external panel for GGA1.

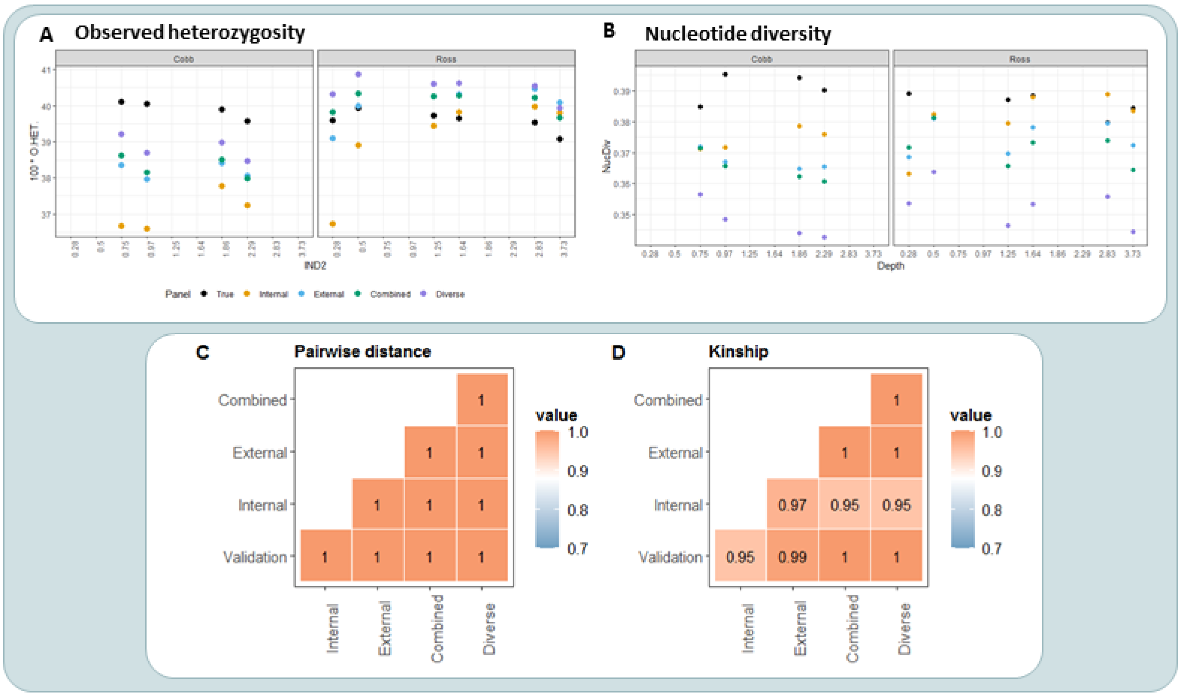

Figure S3. Comparison of the choice of reference panels for 10 validation samples. (A) Observed heterozygosity, (B) nucleotide diversity and correlation plots for (C) pairwise distance and (D) kinship.

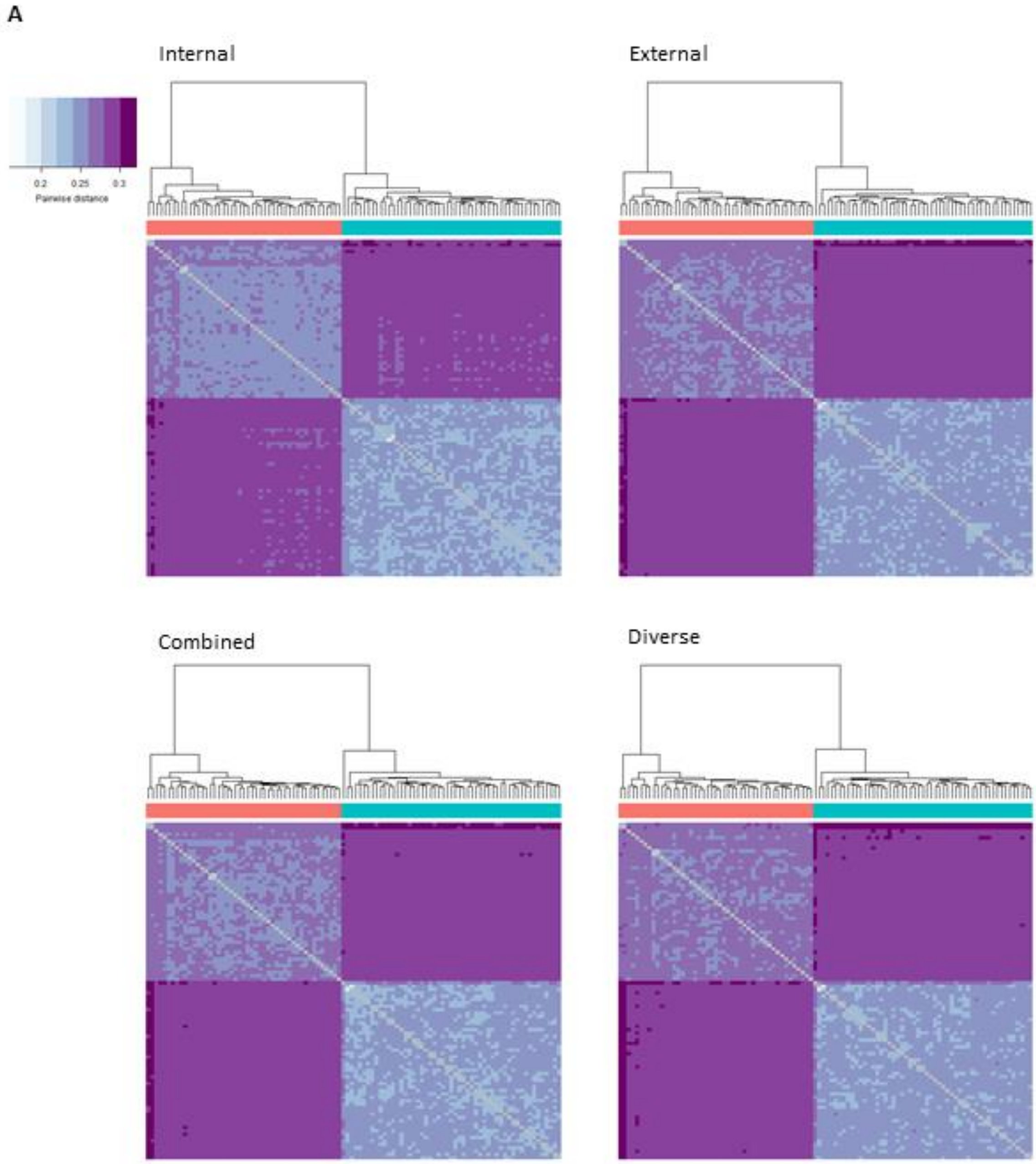

Figure S4. Pairwise distance of the 100 chickens for each reference panel.

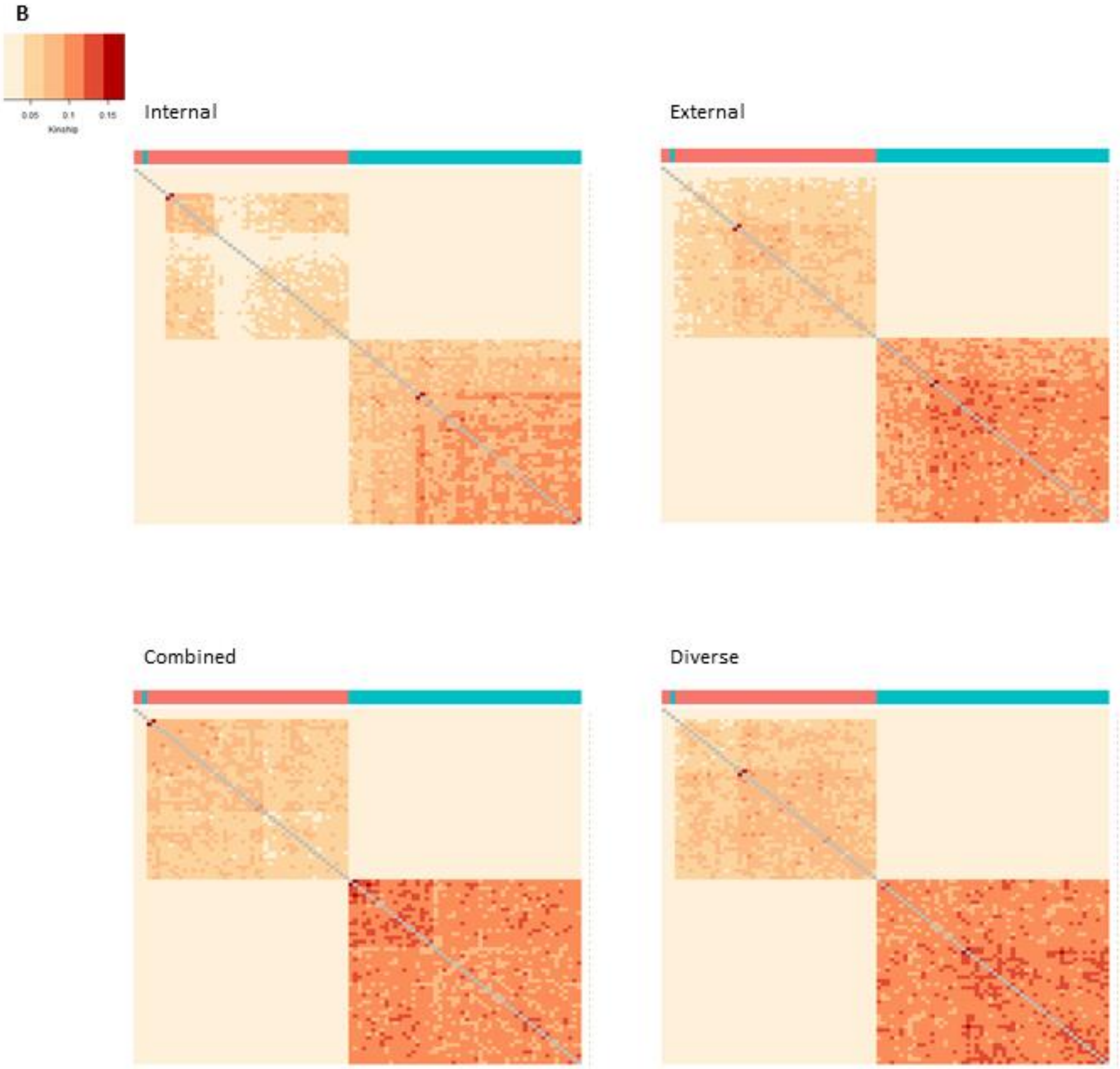

Figure S5. Kinship of the 100 chickens for each reference panel.
