# Supplementary material for: Recovering High‐Quality Host Genomes from Gut Metagenomic Data through Genotype Imputation
Source: Adv Genet (Hoboken). 2022 May 6;3(3):2100065. doi: 10.1002/ggn2.202100065 (PMC9744478; doi:10.1002/ggn2.202100065)
Supplement: Supplementary file 2 — Supplemental Table 1 [file GGN2-3-2100065-s004.pdf]

ADVANCED GENETICS
Open Access

Supporting Information

for *Advanced Genetics*, DOI 10.1002/ggn2.202100065

Recovering High-Quality Host Genomes from Gut Metagenomic Data through Genotype Imputation

Sofia Marcos, Melanie Parejo, Andone Estonba and Antton Alberdi**

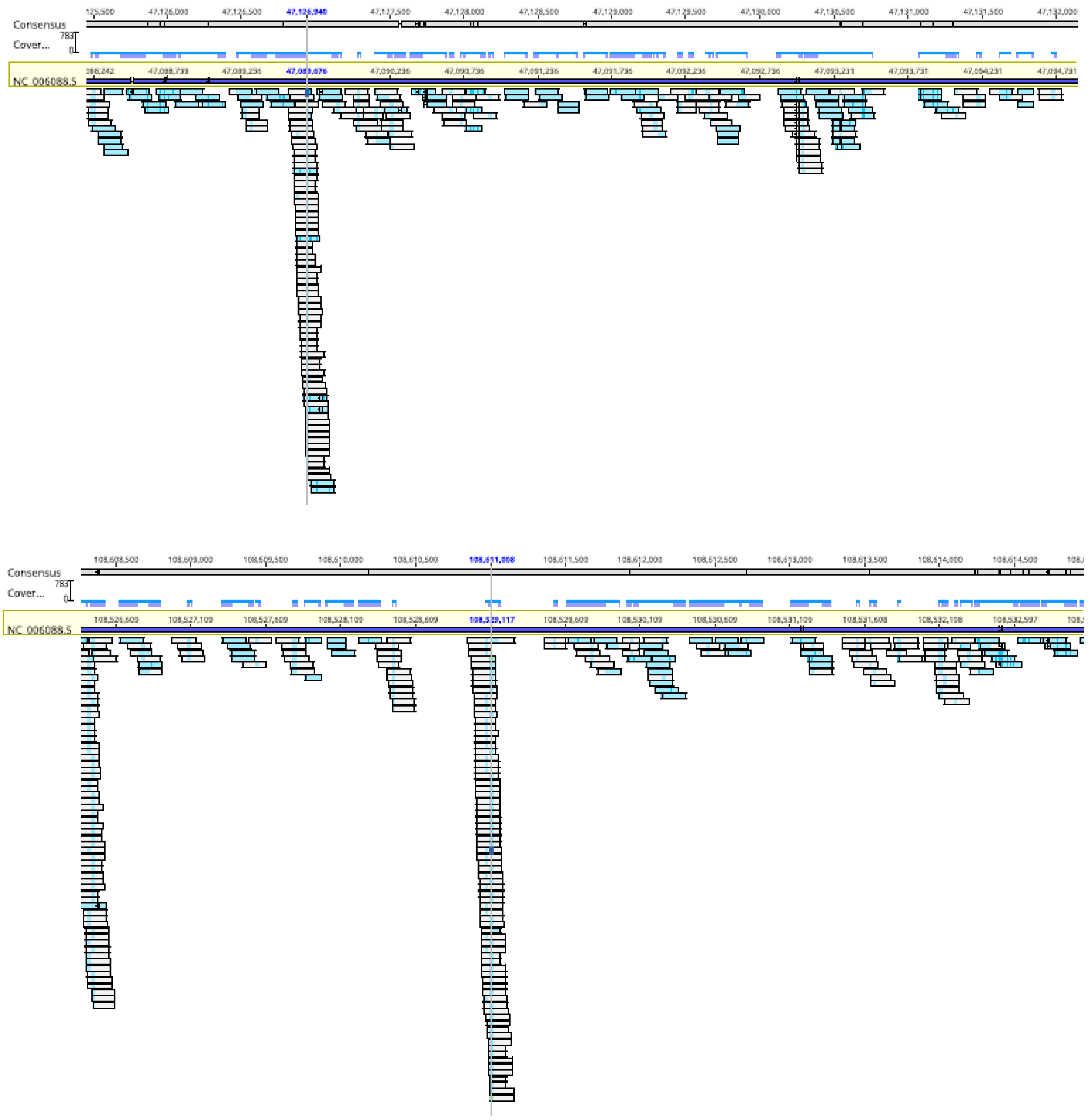

Figure S1. Alignment results before changing seed length from 19 to 25. Captures from 47 Mbp and 180 Mbp regions from GGA1 with Geneious.

Table 1. Mapping depth and breadth results before and after changing seed length from 19 to 25.

**Parameters**	**K19**	**K25**
Depth	2.78	1.73
Std depth	202.79	3.66
Breadth	56.74	51.60
Insert size	20	179

Table 2. Individual mapping depth and breadth of the target population.

**Sample**	**Breed**	**Depth**	**Breadth**	**Host DNA**

1	Cobb	0.33	18.16	1.75
2	Cobb	0.71	35.36	4.71
3	Cobb	0.75	35.92	2.83
4	Cobb	0.75	33.45	3.13
5	Cobb	0.96	47.78	10.23
6	Cobb	1.27	55.76	4.62
7	Cobb	1.28	48.81	10.83
8	Cobb	1.31	56.75	10.55
9	Cobb	1.49	61.06	12.09
10	Cobb	1.56	64.03	20.71
11	Cobb	1.82	67.13	4.29
12	Cobb	1.86	68.77	6.34
13	Cobb	1.86	68.35	15.80
14	Cobb	2.11	72.48	11.75
15	Cobb	2.21	72.34	9.99
16	Cobb	2.23	76.84	29.45
17	Cobb	2.28	73.86	9.51
18	Cobb	2.37	74.82	10.77
19	Cobb	2.67	80.01	15.06
20	Cobb	2.80	78.99	6.82
21	Cobb	3.52	84.73	19.42
22	Cobb	4.41	88.47	16.67
23	Cobb	9.52	95.53	45.16
24	Cobb	0.31	19.50	6.49
25	Cobb	0.32	20.74	5.77
26	Cobb	0.33	22.03	14.22
27	Cobb	0.34	21.39	6.16
28	Cobb	0.35	22.04	5.25
29	Cobb	0.42	25.80	4.51
30	Cobb	0.45	24.83	1.37
31	Cobb	0.47	28.20	3.13
32	Cobb	0.54	29.04	2.29
33	Cobb	0.56	30.14	9.71
34	Cobb	0.58	33.30	4.29
35	Cobb	0.60	32.36	8.06
36	Cobb	0.69	33.19	3.38
37	Cobb	0.72	41.03	11.31
38	Cobb	0.79	43.15	9.63
39	Cobb	1.01	50.37	7.51
40	Cobb	1.57	64.17	21.13
41	Cobb	2.13	68.99	9.94
42	Cobb	2.16	75.88	12.28
43	Cobb	2.79	81.89	62.29
44	Cobb	3.46	87.41	50.47
45	Cobb	5.53	93.00	55.37
46	Cobb	7.56	95.42	43.51

47	Cobb	16.10	96.13	76.26
48	Ross	0.28	14.73	1.13
49	Ross	0.31	18.31	14.06
50	Ross	0.44	24.70	4.13
51	Ross	0.53	30.11	2.75
52	Ross	0.61	34.32	6.99
53	Ross	0.68	34.67	1.96
54	Ross	0.93	45.80	6.31
55	Ross	1.24	54.42	7.14
56	Ross	1.26	56.97	18.60
57	Ross	1.33	55.67	13.05
58	Ross	1.61	61.58	19.05
59	Ross	1.63	62.88	13.16
60	Ross	1.66	63.60	10.40
61	Ross	1.71	63.80	13.53
62	Ross	2.01	66.19	5.98
63	Ross	2.09	67.49	5.64
64	Ross	2.37	74.57	6.60
65	Ross	2.78	80.67	23.45
66	Ross	3.38	83.47	19.26
67	Ross	3.70	89.41	19.29
68	Ross	4.00	86.90	11.35
69	Ross	4.67	90.00	19.06
70	Ross	6.33	93.63	34.86
71	Ross	0.28	18.49	5.51
72	Ross	0.33	21.70	8.89
73	Ross	0.36	19.45	5.03
74	Ross	0.37	23.18	3.93
75	Ross	0.38	24.29	3.19
76	Ross	0.39	25.44	10.15
77	Ross	0.47	29.56	3.74
78	Ross	0.48	28.37	10.45
79	Ross	0.48	29.38	15.4
80	Ross	0.51	29.59	6.21
81	Ross	0.53	32.07	4.56
82	Ross	0.55	30.70	2.86
83	Ross	0.55	30.10	15.26
84	Ross	0.58	35.11	2.62
85	Ross	0.61	33.20	3.38
86	Ross	0.70	37.01	10.59
87	Ross	0.72	41.13	10.73
88	Ross	0.77	38.18	2.96
89	Ross	0.79	43.89	7.63
90	Ross	0.81	43.93	14.67
91	Ross	0.94	48.67	20.36
92	Ross	0.97	48.02	6.64

93	Ross	1.17	54.80	9.73
94	Ross	1.22	59.47	16.06
95	Ross	1.27	56.65	6.57
96	Ross	1.37	60.60	16.53
97	Ross	1.71	68.84	14.39
98	Ross	3.61	88.56	9.42
99	Ross	4.46	91.27	41.04
100	Ross	11.11	96.18	23.4

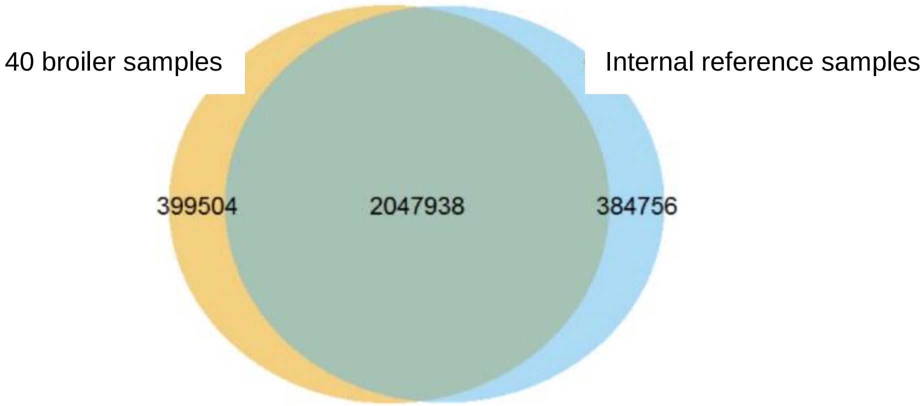

Figure S2. **Venn diagram of shared variants between the internal reference samples and the variant called 40 broilers of the external panel for GGA1.**

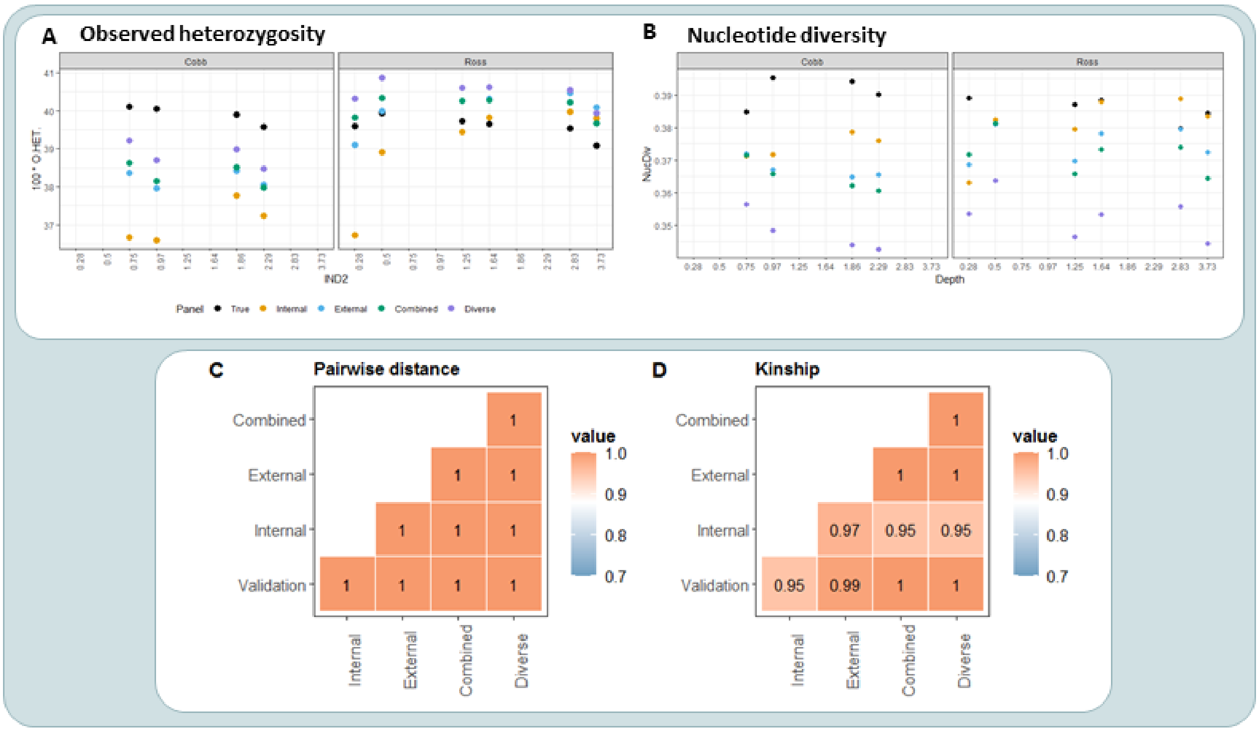

Figure S3. Comparison of the choice of reference panels for 10 validation samples. (A) Observed heterozygosity, (B) nucleotide diversity and correlation plots for (C) pairwise distance and (D) kinship.

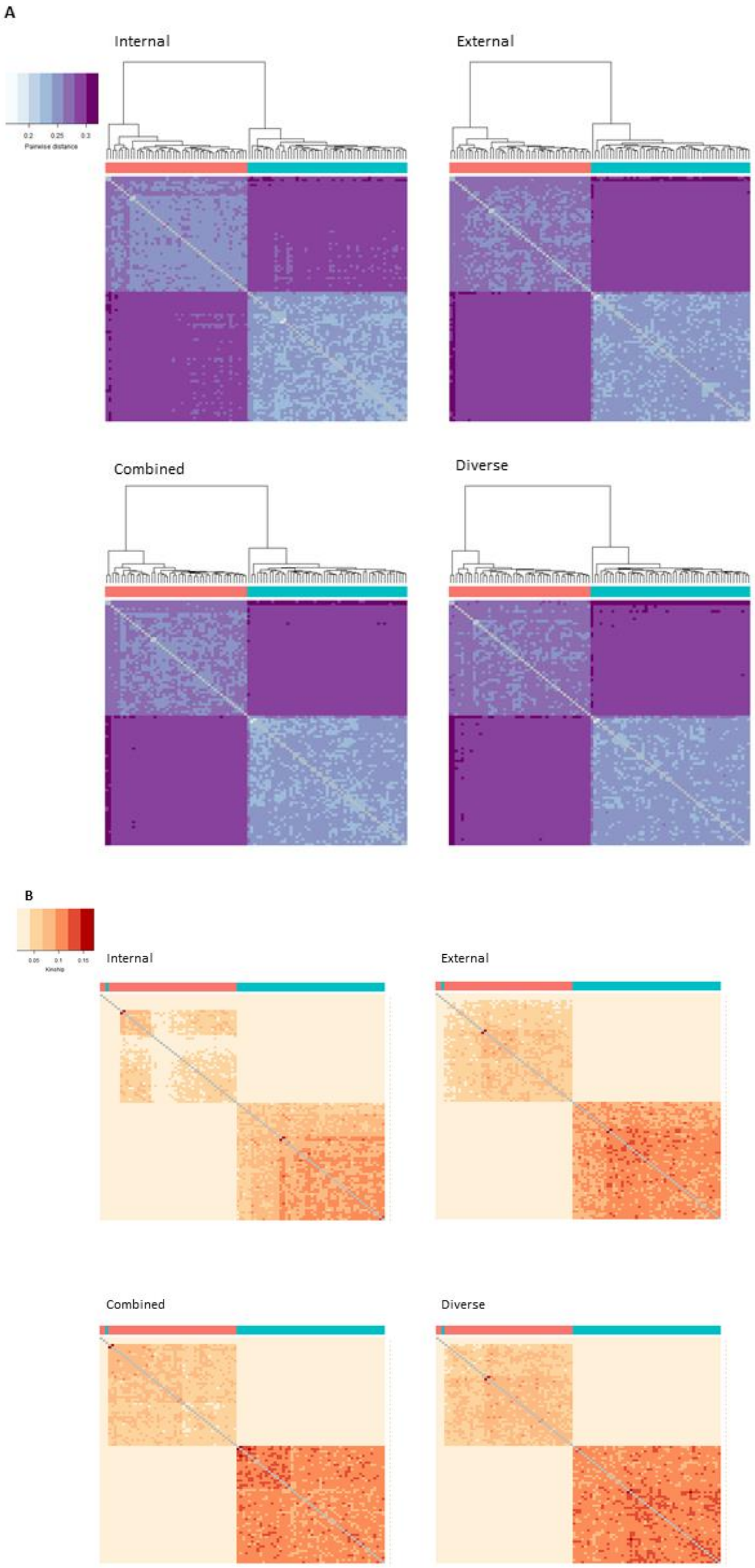
A
Internal
External
0.2
0.25
0.3
Pairwise distance
Combined
Diverse
B
0.05
0.1
0.15
Kinship
Internal
External
Combined
Diverse

Figure S4. (A) Pairwise distance and (B) kinship heatmaps for each of the panels.
